# The perspectives of injection drug users regarding safer injecting education delivered through a supervised injecting facility

**DOI:** 10.1186/1477-7517-5-32

**Published:** 2008-10-29

**Authors:** Danya Fast, Will Small, Evan Wood, Thomas Kerr

**Affiliations:** 1 British Columbia Centre for Excellence in HIV/AIDS, St. Paul's Hospital, British Columbia, Canada; 2 Department of Medicine, Faculty of Medicine, University of British Columbia, Canada

## Abstract

**Background:**

Unsafe injection practices are prevalent among injection drug users (IDU) and have resulted in numerous forms of drug-related harm including HIV/HCV transmission and other bacterial and viral infections. North America's first supervised injection facility (SIF) was established in Vancouver in order to address injection-related harms among IDU. This study sought to examine injection drug users' experiences receiving safer injecting education in the context of a SIF.

**Methods:**

Semi-structured qualitative interviews were conducted with 50 individuals recruited from a cohort of SIF users known as the Scientific Evaluation of Supervised Injection (SEOSI) cohort. Audio recorded interviews elicited IDU perspectives regarding the provision of safer injecting education within the context of a SIF. Interviews were transcribed verbatim and a thematic analysis was conducted.

**Results:**

Participant narratives indicate that significant gaps in knowledge regarding safer injecting practices exist among local IDU, and that these knowledge deficits result in unsafe injecting practices and negative health outcomes. However, IDU perspectives reveal that the SIF allows clients to identify and address these gaps in knowledge through a number of mechanisms that are unique to this facility, including targeted educational messaging that occurs as a part of the drug use cycle and not outside of it, *in situ *demonstration of safer injecting techniques that takes place the moment a client is experiencing difficulties, and enhanced opportunities to seek help from 'expert' healthcare professionals. Importantly, study participants indicated that the overall environment of the SIF promotes the adoption of safer injecting practices over time, both within and outside of the facility.

**Conclusion:**

We conclude that the SIF has been particularly effective in transmitting educational messages targeting unsafe and unhygienic injection practices to a population of active IDU. Consistent with previous work, results of this study indicate that SIFs represent a unique 'micro-environment' that can facilitate the reduction of numerous drug related harms.

## Background

Injection drug use continues to present a major public health challenge in urban settings around the world [1,2]. Unsafe injection practices result in numerous forms of drug-related harm, including overdose [3], HIV/HCV transmission [4,5], and other forms of bacterial and viral infections [6].

Safer injecting education has been widely employed in order to address the harms associated with injection drug use [7,8]. For example, numerous harm reduction programs provide information on safer injecting and street outreach programs work to seek out injection drug users (IDU) in the public venues they frequent in order to provide safer injecting education and other forms of support [9]. However, supervised injection facilities (SIFs) constitute a unique form of intervention, in that they provide a sanctioned drug-using environment that is constantly supervised by healthcare professionals [10].

In September 2003, North America's first SIF, known as "Insite," opened its doors in Vancouver's Downtown Eastside. To date, over 7000 IDU have attended the facility, and approximately 600 injections are supervised at the facility each day [11]. Healthcare professionals are present at all times to supervise injections, intervene in the event of an overdose, and provide safer injecting education.

Previously, a quantitative study was conducted to examine the prevalence and correlates of receiving safer injecting education within the Vancouver SIF [12]. While the results of that study indicated that a significant proportion of SIF users received safer injecting education within the facility, little is known about how safer injecting education is delivered within SIFs, and whether this setting offers advantages over conventional clinic-based or street outreach safer injecting education programs. Further, we know of no studies that have assessed IDU perspectives regarding safer injecting education delivered in the context of SIFs, and there are few qualitative explorations of IDU experiences within SIFs. Therefore, while previous quantitative research has demonstrated the importance of safer injecting education for drug using populations [8,12-15], we aim to extend this work by exploring injection drug users' experiences with receiving safer injecting education in the context of a local SIF.

## Methods

We drew upon data from 50 in-depth qualitative interviews conducted from November 2005 to February 2006. Interviewees were recruited from the Scientific Evaluation of Supervised Injecting (SEOSI) cohort, which is composed of over 1000 randomly selected SIF users in Vancouver [16]. Interview participants were selected on a daily basis from persons attending the research office for quantitative cohort interviews. Recruiting efforts intentionally created a sample composed of individuals with differing levels of SIF utilization that was also representative of the local injecting population in terms of gender, age, and ethnicity (see Table 1). Interviews were undertaken by three trained interviewers (two male and one female) and facilitated through the use of a topic guide encouraging discussion of SIF use and experiences with receiving safer injecting education within the facility.

**Table 1:** Characteristics of qualitative study sample compared to a representative sample of SIF clients (SEOSI)

	**Qualitative Interview Participants**	**SEOSI Cohort**
**Total Number**	50	1090
**Median Age **(range)	38 (25–60)	38.4 (18.9–63.7)
**Gender**		
Female, n (%)	21(42)	313(29)
Male, n (%)	28(56)	773(71)
Trans-gendered, n (%)	1 (2)	4 (< 1)
**Aboriginal Ethnicity**		
Yes, n (%)	13(26)	211(19)
No, n (%)	37(74)	879(81)

Interviews lasted between 30 and 60 minutes, were tape-recorded, and were later transcribed verbatim. The content of transcribed interviews was reviewed, and all text segments (both positive and negative) related to safer injecting education received within the SIF were catalogued. The catalogued data was subsequently subjected to a thematic analysis which focused on the social processes and characteristics of the SIF which were reported to influence experiences with safer injecting education.

All participants provided informed consent to participate, and the study was undertaken with appropriate ethical approval granted by the Providence Healthcare/University of British Columbia Research Ethics Board. Participants were compensated for their time with a twenty dollar honorarium. There were no refusals of the offer to participate in the interview, and no drop-outs occurred during the interview process.

## Results

The sample of qualitative interview participants was composed of 21 women, 28 men and one trans-gendered individual. The age of participants ranged from 25 years to 60 years, and the median age of participants was 38. Excerpts from the qualitative interviews are presented below in order to illustrate the central themes that emerged in the analysis. Considerable overlap was observed across thematic areas.

Participant narratives indicate that significant gaps in knowledge regarding safer injecting practices exist among local IDU; however, study participants also indicated that the SIF allows clients to identify and address these gaps in knowledge through a number of mechanisms that are unique to this facility.

### 1. Gaps in knowledge

Several participants articulated a general lack of knowledge regarding safer injecting practices prior to using the SIF. This lack of knowledge was not limited to new injectors:

*R: I learned one thing in there that I didn't know after twenty-three years of using and that's the bevel side up! Y'know, with your, with your, ah, syringe. *(Female Participant #11)

*R: I think that since the injection site has been open I've learned how to inject properly . . . and there were things I never even knew, in all the whole 20 years I've been shooting dope. *(Female Participant #44)

Participants connected gaps in knowledge and improper injecting techniques with negative health consequences, such as scarring or repeated injection-related infections such as abscesses:

*R*: [Before using Insite] *I wasn't cleaning *[my skin]. *I wasn't – the needle wasn't facing up. I was just sticking it in anywhere, poking holes in myself. Using dull needles. Using the same one over and over again. Not tying it off. Not doing anything properly*.

I: Can I just ask: how long have you been fixing for?

*R: I'm twenty seven. The first drug I ever did was heroin, and it was in a needle at eleven years old. And I've been using steadily since I was twelve, but I still use it a lot*.

*I: So that's a long time to go without really knowing how to inject properly*.

R: *Yeah. Yeah. Real long time. That's why I have all the scars I do ... *(Male Participant #27)

In a number of cases, participants were not aware that they were injecting unsafely until they began using the SIF and receiving safer injecting education from on-site healthcare professionals. Visits to the SIF allowed participants to identify and address specific gaps in knowledge, resulting in the adoption of safer injecting practices and improved health outcomes in several cases:

*R: I learned how to fix myself properly in there . . . I think it's had an affect on, well, I know it has *[had an effect on my health] *because I know how to inject properly now. And I know it has for other people, too. *(Female Participant #11)

*R: Well, when I first went there *[to the SIF]* I used to just take my dope and shake it, and they *[the nurses]*  said, "Well, you should be cooking it because it takes all the impurities out. And you should use an alcohol wipe, like, to sterilize your --"*

*I: Yeah, clean up your skin or whatever*.

*R: And I never used to do that. And since I've been doing that, and since they showed me the right way, I haven't had any abscesses. So, I credit them with that. *(Female Participant #30)

### 2. Addressing gaps in knowledge

#### A. Educational messages delivered within a sanctioned drug-using environment

Despite the availability of safer injecting education via a number of other service providers in locations throughout the Downtown Eastside, several participants noted that the SIF was the first place that they had successfully been able to receive help:

I: Okay, so where would you be getting this help if you weren't getting it from Insite?

*R: I've never looked at *[getting help] *before Insite so, I don't know . . . *(Male Participant #39)

*I: What would you have to say about the help that you've got *[at Insite]*?*

*R: I think it's very fair, and it's sufficient . . . I can't really compare it to anything else 'cause I've never really gotten any help anywhere else, other than there. *(Trans-gendered Participant #2)

Participants articulated the benefits of receiving educational messages within a sanctioned drug-using environment that is a part of the drug using cycle and not outside of it. Multiple visits to the SIF for the primary purpose of consuming drugs meant that educational messages were both highly accessible and reinforced over time. Receiving regular safer injecting education during the process of consuming drugs was contrasted with receiving educational messages at other times, when priorities associated with a lifestyle of addiction – such as obtaining drugs or obtaining the money to buy drugs – would likely outweigh the perceived need to access assistance from healthcare professionals:

*R: Being an addict . . . you do want to go out and utilize the *[service]* facilities, but if it's *[on the] *way y'know? *[If]* it's not on the way to go get your money to get better *[i.e. use drugs], *and once you get better, you got to go get more money for the next day or whatever, but if something's right then and there then it's like perfect. *(Female Participant #38)

#### B. Enhanced opportunities to seek help from healthcare professionals

Multiple visits to the SIF for the primary purpose of consuming drugs was also said to facilitate the development of meaningful relationships with 'expert' healthcare professionals. For those participants who had not been able to access help previously, SIF nursing staff represented an essential source of reliable and accurate information. The safer injecting education received at the SIF from healthcare professionals was contrasted with educational messages or information one could pick up 'on the street' from other IDU:

*I: You know a lot about how to inject safely, it sounds like. Is this stuff you knew before *[using Insite]*?*

*R: No, I didn't . . . I always had to have someone *[inject]* it for me, so, most of this, most of the information that I got is just from people *[on the street] . . . *from Insite, you know, like*, [I learned] *the proper way I guess. You know, a lot of people *[on the street]* don't really tell you about the cleaning or using alcohol swabs and stuff like that. They always neglect to leave that stuff in, put that stuff in when they're telling you *[how to inject]. *Insite was where I learned that you should use the alcohol swabs. I didn't know that before . . . *(Trans-Gendered Participant #2)

While it was acknowledged that one's peers could not always be relied upon to provide complete educational messages, SIF nurses were viewed as experts who could be trusted to deliver accurate and comprehensive safer injecting education:

*R: I mean you have people *[i.e. nurses]* who are experienced. You have people who know what they're talking about, what they're doing, showing you how to do it properly, how to do it safely, how to do it cleanly . . . She's generally smarter than the rest, y'know. I mean, when you have medical questions, she's the one to ask ninety percent of the time. *(Male Participant # 27)

The SIF was described as providing a context in which on-site healthcare professionals are able to guide clients through each step of the safer injecting process at a comfortable pace, and often over the course of multiple interactions. Healthcare professionals are able to tailor educational messages to suit the specific needs of each client to address specific deficiencies in practice, and are able to intervene as a client is experiencing difficulties. Participants valued the non-judgmental attitudes of the SIF staff, and felt able to ask questions and raise concerns the moment they thought of them:

*R: Like, if you ever are curious or just need information, you know it's there, and you can ask for it and get it. You don't have to wonder at all. *(Male Participant #40)

*R: Sometimes there's a point where I asked *[the nurse], *"I'm having troubles getting a vein here, so is there somewhere else that I can *[inject myself]*?" And she, y'know, she showed me a few places. *(Female Participant #21)

#### C. *In situ *demonstration of safer injecting techniques

Many respondents articulated the benefits of being *shown *specific safer injecting techniques during the actual process of injecting, as opposed to simply being told how to inject properly, or provided with more general printed educational materials at another time:

*R: I remember one time asking the nurse just to give me a kind of a more visual way – showing me how to tie-off and stuff – and she gave me some pointers. *(Male Participant #9)

*R: If I'm at Insite they can show me where there's a vein and bang! – I got it instantly and I don't gotta sit there you know, with a needle for half an hour, you know, blood coming everywhere and that. *(Trans-Gendered Participant #2)

Several participants indicated that they had required help injecting prior to using the SIF, or that they sometimes continued to require help injecting when they were using drugs in locations other than the SIF. In some cases, safer injecting education received within the SIF – and specifically, *in situ *demonstration of how to locate a viable vein – was connected with developing the skills required to inject more independently:

*R*: [Bevel facing up] *was one simple, little thing I didn't realize . . . So bevel up and I'm able to get myself almost every single time, and I always needed a doctor *[i.e. someone who performs assisted injections]. *Like, my partner always had to inject me, and that was really frustrating. *(Female Participant #11)

*R: They have a nurse there that's qualified, and can show me, and you know, point *[veins]* out and help me*.

I: Ok, so you can usually fix yourself, because of the help that they've given you?

*R: Yes, yes*.

*I: Ok, and do you think that if you, in those situations *[when you can't easily find a vein], *if you weren't injecting at Insite, you would be getting help *[injecting] *from somebody else?*

*R: Yes. *(Trans-Gendered Participant #2)

Those who were adamant about *avoiding *assisted injection also articulated the benefits of *in situ *demonstration of safer injecting practices to address specific gaps in knowledge:

*R: Y'know, I got a hole in my arm. It's just healing, I've had plastic surgery on it and, I, I fix in it all the time. Y'know*, [the nurses at the SIF] *say, "Don't use that! Maybe use your leg," so the one lady showed me on the doll, showed me where the veins go up your leg, here. Showed me how to, y'know, "Try there, use a tie", 'cause I never used to use a tie . . . And it's really helped, 'cause now I can use that. It works very well, right? . . . Yeah, 'cause I won't let anybody fix me, right? *(Female Participant #12)

In general, the safer injecting education received at the SIF enabled some participants to take greater control of their injecting practices, at least within the SIF.

### 3. An overall environment that promotes safer injecting practices

Even when participants had accessed safer injecting education prior to using the SIF, it was noted that the overall atmosphere of the SIF made them more conscious of these messages, and more diligent about putting them into practice:

*I: What do you mean when you say you're more aware when you inject *[at Insite]*?*

*R: I dunno. Just more aware – there could be health issues involved. Like, if I didn't use the alcohol swab, I could get an infection. If you use a rig more than once, it could be dull and it could hurt, or it could get bacteria on the end of it. Y'know, like, you can give yourself *[an] *infection which really boggled my mind the first time I'd heard it . . . It just makes you more aware that there's a process that can be beneficial to everybody, right?*

I: Are any of those things that you heard for the first time when you were at Insite?

*R: No, I don't think so. It just made me more conscious, self-conscious of it. *(Female Participant #32)

Participants reported that the provision of sterile syringes and the necessary ancillary injecting equipment, combined with the provision of targeted, *in situ *safer injecting education by trusted experts, all served to reinforce educational messages and contribute to an overall atmosphere that encourages the adoption of safer injecting practices:

*R*: [Insite is]* safe because it's constantly supervised. Everything is clean. Sanitary, hygienic, whatever. All the supplies, obviously . . . If you need any guidance or whatever, there's always a nurse on-hand. *(Female Participant #32)

*R: When I go in there I'm more self-conscious; like, you got your tie-on there, you got your Band-aids, you got your water, and you got your filter . . . There I always go through the whole process properly. So, I should go there all the time. *(Male Participant #9)

Importantly, IDU narratives reveal that once safer injecting habits are established within the SIF, it becomes more likely that they will practice safer injecting techniques outside of the SIF as well:

*R: People are being safer and everything too, eh? It's... y'know, as I say, heroin addicts especially are creatures of habit. They go in there, they get the habit formed of being safe, they'll use the same quality when they go out on the street. "Oh, do you have a tie-off? Do you--?" Y'know, they'll make sure. . . They'll carry on the same values that are drilled into them in there *[at the SIF]. (Male Participant #6)

## Discussion

In sum, participant narratives indicate that significant gaps in knowledge regarding safer injecting practices exist among local IDU, and that these knowledge deficits result in unsafe injecting practices and negative health outcomes for numerous local IDU. However, IDU perspectives reveal that the SIF allows clients to identify and address these gaps in knowledge through a number of mechanisms that are unique to this facility, including targeted educational messaging that occurs as a part of the drug use cycle and not outside of it, *in situ *demonstration of safer injecting techniques that takes place the moment a client is experiencing difficulties, and enhanced opportunities to seek help from 'expert' healthcare professionals. Importantly, study participants indicated that the overall environment of the SIF – including the provision of comprehensive sterile injecting paraphernalia and the constant presence of healthcare professionals – promotes the adoption of safer injecting practices over time, both within and outside of the facility. Interestingly, we found little variation in experiences with receiving safer injecting education within the SIF according to gender, age or ethnicity, in spite of evidence which indicates that women may be more likely to require help injecting for a variety of contextual reasons [17-19], and may therefore be more receptive to assistance and education from on-site healthcare professionals.

IDU utilize the SIF for the primary purpose of consuming drugs; however, multiple visits to the facility give nurses the opportunity to provide hands-on, client-centered safer injecting education in a timely and unhurried manner. Within the SIF, *in situ *demonstration of targeted educational messages can occur the moment a client is experiencing difficulties, and at a pace that is acceptable to the client. In combination with the provision of sterile syringes and the necessary ancillary injecting equipment, this process encourages clients to reflect on and enact safer injecting practices without feeling rushed. This can be contrasted with the circumstances that often surround public injection, where exposure to police scrutiny and the possibility of arrest often results in skipped steps and unsafe injecting behavior [20-24], despite the availability of safer injecting education and sterile injecting paraphernalia in public venues via street outreach approaches.

Ongoing drug-related harms among IDU indicate that novel public health interventions are needed [19,25]. Educational approaches have typically been based on the assumption that IDU are autonomous actors operating within relatively stable social environments [26]. Even those street outreach approaches that seek out IDU in the public venues they frequent oftentimes fail to recognize the macro- and micro-environmental factors that limit an individual's ability to initiate behavioral change [27,28]. In order for educational approaches to be effective, attention must be paid to the physical and social environment that influences the production of safety and risk for individuals who use injection drugs [27,29]. Broader conceptualizations of risk, such as Rhodes' 'risk environment' framework [28], call for structural and environmental interventions that alter aspects of the context in which injection drug use occurs, thereby facilitating the adoption of safer injecting practices and the reduction of drug-related harms [30]. The results of this study indicate that SIFs represent one such micro-environmental intervention with a unique social composition that serves to modify risk in ways that differ from conventional clinic-based and street outreach educational approaches. As such, SIFs have significant potential to facilitate the adoption of safer injecting practices where other educational approaches have failed or been less effective.

The results of this study indicate that, by addressing critical gaps in knowledge, SIFs can foster greater injecting independence among IDU and contribute importantly to the reduction of injection drug-related harms. Previous research has noted that a lack of knowledge regarding how to safely inject oneself is a primary explanation for requiring help with injecting [31], and that requiring help with injecting places individuals at heightened vulnerability for HIV and HCV infection [13]. These observations were reflected in IDU narratives that emphasized the importance of timely and appropriately delivered safer injecting education for individuals who engage in assisted injection as a result of lack of knowledge. The safer injecting education received at the SIF allowed some study participants to take greater control of their injecting habits. The fact that a previous quantitative study found that requiring help injecting was independently associated with receiving safer injecting education at the Vancouver SIF [12], combined with the results of this qualitative analysis, provides good indication that safer injecting education within the SIF may have significant implications for HIV and HCV prevention among frequent SIF users.

The present study has several limitations that warrant acknowledgement. Firstly, our findings are based upon interviews with local IDU participating in the current study. While an effort was made to ensure that the study sample reflects the demographics of the local SIF-using population, some perspectives may nonetheless be underrepresented. Secondly, although interviewees were told that the study was being conducted independently of the SIF, it is possible that social desirability bias affected the responses of some participants. Thirdly, the data collected and analyzed here presents only the viewpoints of IDU; the results of this analysis should be compared with the findings of ethnographic research utilizing participant-observation within the SIF. Interviews with healthcare professionals and other SIF staff should be conducted to elicit alternative perspectives. Fourthly, it must be recognized that the Vancouver SIF is not accessible to all local IDU; people who rely on others to administer all injections (such as IDU with physical disabilities), or who engage in assisted injection for a variety of socio-cultural reasons [32], are excluded from the facility as a result of regulations prohibiting assisted injection within the SIF. Thus, these individuals are not able to receive safer injecting education via the SIF, limiting the facility's effectiveness in addressing the needs of diverse local IDU. Furthermore, while those participants who had received safer injecting education within the facility reflected positively on their experiences, a minority of participants reported that they had not received safer injecting education within the facility, or that they disliked the overall environment within the facility (which would likely discouraged them from spending extra time necessary to seek out and engage in safer injecting education with on-site healthcare professionals). The results of this analysis suggest that the SIF can greatly benefit those individuals who visit the site regularly and have developed good relationships with on-site healthcare professionals; future research is needed to determine why some individuals use the facility more infrequently than others, and how this affects the uptake of safer injecting education.

In summary, the results of this study indicate that the SIF has been effective in transmitting educational messages targeting unsafe and unhygienic injection practices to a population of active IDU. The SIF facilitates the delivery and adoption of educational messages via a number of mechanisms that are unique to this facility and highly acceptable to local IDU. Consistent with previous work, results of this study indicate that SIFs represent a micro-environmental intervention with significant potential to reduce numerous drug-related harms. Importantly, this study contributes to the development of knowledge regarding alternative mechanisms of connecting IDU with safer injecting education.

## Competing interests

The authors declare that they have no competing interests.

## Authors' contributions

WS and TK designed the study. TK and DF conducted the analyses of the data. DF prepared the first draft of the article. All authors contributed to the revision of the manuscript.
